# Bacterial genotoxin functions as immune-modulator and promotes host survival

**DOI:** 10.15698/mic2016.08.520

**Published:** 2016-07-28

**Authors:** R Guidi, L Del Bel Belluz, T Frisan

**Affiliations:** 1 The Laboratory of Allergy and Anti-Helminth Immunity, The Francis Crick Institute, London, UK.; 2 Dept. of Cell and Molecular Biology, Karolinska Institute, Stockholm Sweden.

**Keywords:** genotoxin, Salmonella, typhoid toxin, DNA damage, inflammation, persistent infection

## Abstract

Bacterial genotoxins are effectors that cause DNA damage in target cells. Many aspects of the biology of these toxins have been characterised *in vitro*, such as structure, cellular internalisation pathways and effects on the target cells. However, little is known about their function *in vivo*.* Salmonella enterica* serovar Typhi (S. Typhi) is a Gram-negative, intracellular bacterium that causes typhoid fever, a debilitating disease infecting more than 20 million people every year. *S. * Typhiproduce a genotoxin named typhoid toxin (TT), but its role in the contest of host infection is poorly characterized. The major obstacle in addressing this issue is that *S. * Typhi is exclusively a human pathogen. To overcome this limitation, we have used as model bacterium *S.* Typhimurium, and engineered it to produce endogenous levels of an active and inactive typhoid toxin, hereby named as TT (or genotoxic) and *cdtB* (or control), respectively. To our surprise, infection with the genotoxin strain strongly suppressed intestinal inflammation, leading to a better survival of the host during the acute phase of infection, suggesting typhoid toxin may exert a protective role. The presence of a functional genotoxin was also associated with an increased frequency of asymptomatic carriers.

 Bacterial genotoxins are proteins and metabolic products secreted by pathogenic and commensal bacteria. Our laboratory has studied for a long time the cytolethal distending toxin (CDT), produced by several Gram negative bacteria, such as *Escherichia coli, Campylobacter sp*. and *Helicobacter sp.* Once released from the producing bacterium, CDT binds to the cell surface via glycosylated-receptors, and triggers its internalisation and retrograde transport towards the endoplasmic reticulum (ER). From the ER, CDT uses a non-classical Endoplasmic Reticulum Associated-protein Degradation (ERAD) pathway to enter the nucleus, where its catalytic subunit induces DNA breaks. CDT intoxication triggers the classical DNA damage response, resulting in cell cycle arrest and DNA repair activation. Incomplete DNA repair will eventually promote apoptosis or acquisition of a permanently quiescent status, known as senescence. Similar DNA-damage activity is reported for the typhoid toxin and the genotoxin colibactin (produced by *E. coli*). Differently from CDT, which is mainly produced by extracellular bacteria, the typhoid toxin is produced upon bacterial internalisation and replication within the *Salmonella* containing vacuole (SCV), inside the host cell. In S. typhimurium, the toxin is secreted by the producing bacterium within Outer Membrane Vesicles (OMVs), which accumulate within the SCV and are released into the extracellular milieu via an exocytosis-like process. In *S.* Typhi, it is also being shown that typhoid toxin require a special N-acetyl-β-D-muramidase to escape the periplasm and to be secreted in the cellular space.

*S.* Typhi is a higher-primate restricted pathogen that spreads through contaminated food and water. Between 1 to 6% of infected individuals never eradicate the infection, becoming chronic asymptomatic carriers, and epidemiological studies showed that these individuals are at high risk to develop hepatobiliary and gallbladder carcinoma. Due to its ability to directly damage cellular DNA, typhoid toxin could contribute to the carcinogenic properties of *S.* Typhi. The role of bacterial genotoxins as potential carcinogens is supported by the demonstration that long-term exposure to CDT *in vitro* promotes: i) genomic instability, ii) alteration of the DNA damage response, iii) activation of survival signals, iv) acquisition of carcinogenic traits. Furthermore, a higher percentage of *E. coli* isolates expressing colibactin and CDT have been detected in the mucosa of colorectal cancer (CRC) and Inflammatory Bowel Disease (IBD) patients compared to control subjects.

Infection with CDT producing *H. hepaticus* and *C. jejunium* promotes gastric or intestinal colonization, enhances inflammatory response, and development of hepatic dysplastic nodules in several mouse models, while intravenous injection of purified typhoid toxin recapitulates the effect of typhoid fever in mice. It is noteworthy that most of these *in vivo* studies have been performed in immune deficient mice or using toxin delivery routes that do not mimic the natural course of the bacterial infection.

These data prompted us to develop a model to study the biological function of typhoid toxin in acute and chronic *Salmonella* infection in an immune competent host.

To this end, we engineered an *S.* Typhimurium strain, which causes systemic typhoid fever-like infection in mice but does not naturally harbors the toxin. We inserted the operons encoding for typhoid toxin genes into the bacterial genome, and, as control, we produced a *S.* Typhimurium strain containing the toxin operon lacking the gene of active subunit (∆*cdtB*). Both strains were able to infect cells *in vitro*, and a robust induction of DNA damage was readily detected in cells infected with the genotoxic strain 24h after infection.

**Figure 1:**
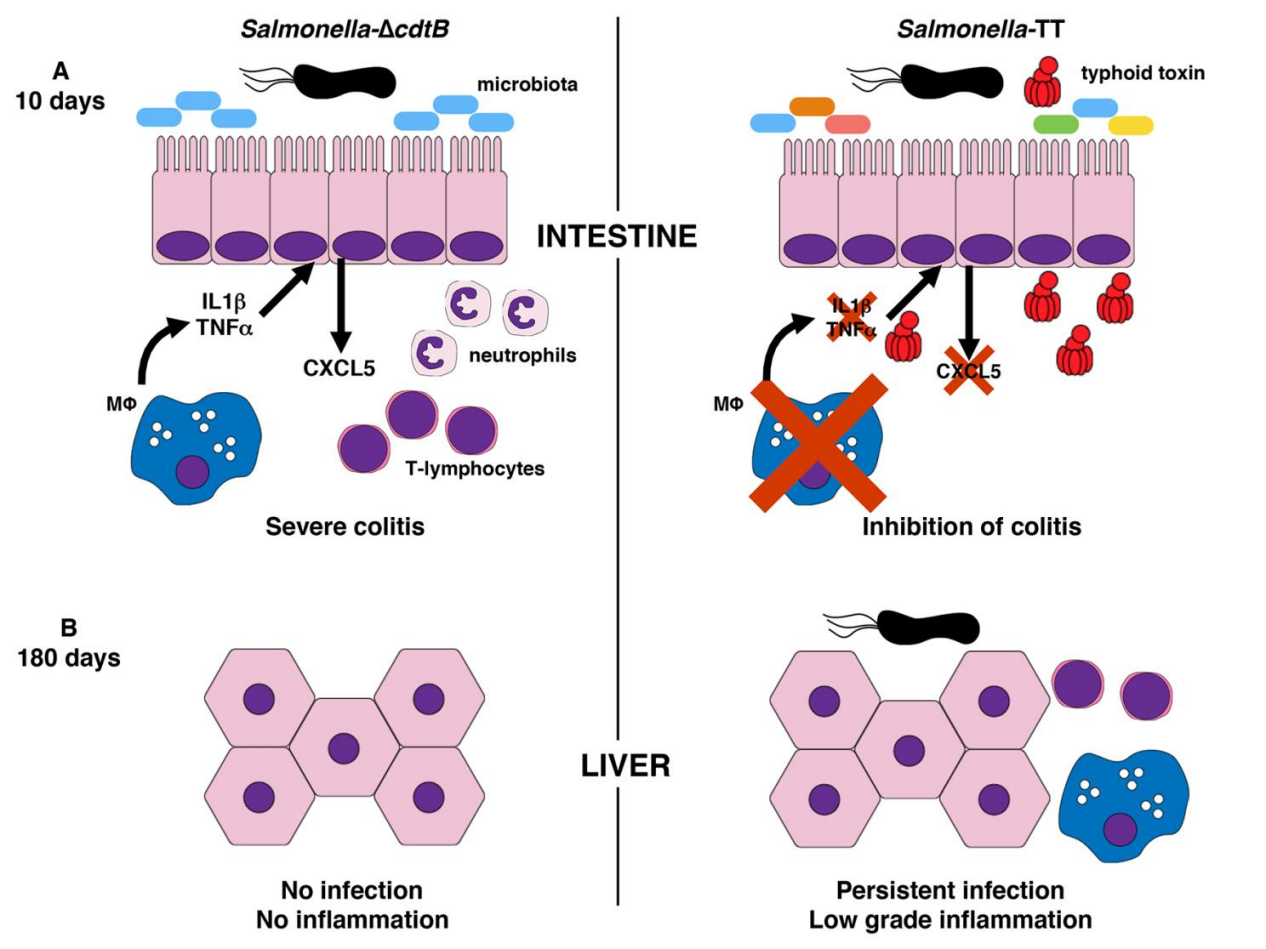
FIGURE 1: Schematic representation of typhoid toxin function *in vivo*. **(A) Intestine.** Mice infected for 10 days with the control Δ*cdtB S*. Typhimurium (left panel) present severe intestinal inflammation, characterized by the recruitment of neutrophil, macrophage and T lymphocytes at the lamina propria. Transcripts levels of pro-inflammatory cytokines (e.g. *Tnfa *and *Il1b*) and chemokines (*Cxcl5*) are also increased compared to the levels observed in mice infected with the genotoxic strain (right panel). The reduced inflammatory environment in mice infected with the genotoxic strain is associated with a different composition of the gut microbiome. **(B) Liver.**At 180 days post-infection, the presence of the typhoid toxin promotes the establishment of a chronic asymptomatic infection, associated with a low-grade hepatic inflammation, characterized by infiltration of macrophages and T lymphocytes, and increased mRNA expression of pro-inflammatory mediators.

##  Infection with the toxigenic S. Typhimurium strain enhances host survival and promotes persistent infection

The mouse model of choice was the sv129 strain, which does not easily succumb to *Salmonella* infection and it is preferred for the study of long-term infections. Three groups of mice were either mock infected or infected with the toxigenic and control *S.* Typhimurium strains for 10, 30, 60 and 180 days. Presence of the active genotoxin subunit cdtB was detected in the nucleus of hepatic cells in mice infected with the TT strain for 10 days, indicating that, in these experimental conditions, the toxin is expressed *in vivo*. 

According to the literature data, 40% of the mice infected with the control strain died within 15 days post-infection. Surprisingly, all mice infected with the genotoxic strain survived this critical period. This effect was not due to an impaired invasive capacity of the TT strain compared to the control ∆*cdtB Salmonella*, since we recovered equal amount of bacteria from all the organs of the infected mice, independently of the strain used. Once this critical window of time was overcome, all mice survived until the end of the experiment, which allowed us to study the long-term infections. Interestingly, the presence of the genotoxin was associated with the establishment of a persistent infection, since we could recover bacteria from the liver of 83% of the mice infected with the TT strain 180 days post-infection, while no bacteria were isolated from mice infected with the control strain.

## Typhoid Toxin as organ-specific immune modulator

Histological and transcriptomic data revealed a strong inflammatory response in mice infected with the ∆*cdtB* strain, characterised by neutrophils infiltration and recruitment of macrophages and T lymphocytes in the lamina propria. These effects were greatly reduced in the TT-infected mice, where we found lower levels in inflammatory infiltrates, reduced transcriptional levels of macrophage markers and of *Cxcr5*, a chemokine that mediate neutrophils recruitment. We also found a reduced transcriptional profile associated with activation of a T helper (Th) 1-mediated response. The observation that neutrophils are greatly reduced in TT-infected mice is in line with previous observation, demonstrating a decrease of polymorphous lymphoid cells (PMLs) from the blood of mice upon intravenous injection of purified toxin. Thus, the increased survival rate of mice infected with the genotoxic strain was associated with a strong reduction of the severe enteritis.

Upon entry through the intestinal mucosa, *Salmonella* spreads to colonise the hepato-biliary system, which represents the site of persistent infection in asymptomatic chronic carriers. 

At the early phase of infection, we did not detected any significant difference in the inflammatory response in the liver of mice infected with the genotoxic strain compared to the response observed in mice infected with the control *Salmonella*. However, the establishment of a persistent infection in the liver of mice infected for 180 days with the genotoxic strain was associated with an increase in T lymphocyte and macrophage infiltration, and higher mRNA levels for gene sets associated with interferon and NFκB signaling, transcription factors, and Th1 responses. 

These data suggests a dualistic role for the typhoid toxin, depending on the target organ: it facilitates a stealthy intestinal invasion, suppressing the host inflammatory response, yet causes a low liver inflammation in asymptomatic chronic carriers, which may contribute to hepatobiliary cancer development when the tissue homeostasis is altered by conditions such as acute or chronic liver disease.

## Typhoid Toxin as modulator of the gut microbiota

Chronic inflammation of the digestive system (including hepatitis) is accompanied by changes in the intestinal microbiota that may exacerbate the inflammatory response, and delayed its resolution. To study whether infection with the genotoxic strain had an impact on the intestinal ecosystem, we sequenced 16S rRNA genes from stool samples from mice during early (day 10) and late (day 60 and 180) period post-infection. We found that the presence of the typhoid toxin was associated with increase in mutualistic components of the intestinal microbiota, such as *Bacteroidaceae*,* Clostridiales* and *Deferribacteraceae*, while infection with the control strain was associated with a reduction of families that exert anti-inflammatory effects (*Lactobacillaceae* and *Lachnospiraceae*), resulting in a pro-inflammatory configuration of the gut ecosystem.

## Conclusion

In summary our data demonstrated that the presence of the typhoid toxin reduces severe colitis, allows a stealthy infection, a better chance to survive the acute phase of the disease, and further promotes the establishment of an asymptomatic carrier. Considering that *S.* Typhi is an exclusive human pathogen, this strategy would allow the maintenance of the bacterial reservoir, allowing replication, survival and spread.

In this context, the typhoid toxin would resemble more an immune-modulatory molecule rather than a toxic agent.

